# Standard manual capsulorhexis / Ultrasound phacoemulsification compared to femtosecond laser-assisted capsulorhexis and lens fragmentation in clear cornea small incision cataract surgery

**DOI:** 10.1186/s40662-016-0050-x

**Published:** 2016-07-29

**Authors:** Anastasios John Kanellopoulos, George Asimellis

**Affiliations:** 1 Laservision.gr Clinical and Research Eye Institute, Athens, Greece; 2 Department of Ophthalmology, NYU Medical School, New York, NY USA

**Keywords:** Femto-second laser cataract surgery, Manual capsulorhexis LenSx, Toric IOL, Phacoemulsification, Refractive outcomes, Endothelial cell counts, Corneal edema, Clear cornea small-incision cataract surgery

## Abstract

**Background:**

Femtosecond-laser assisted clear cornea cataract surgery may hold promise in safer and more effective procedures. We decided to perform a comparative study to standard manual incision phacoemulsification surgery.

**Methods:**

This is a single-center, single-intervention, and prospective comparative data evaluation of 133 consecutive cases subjected to cataract surgery. Group-A (Phaco), manual capsulorhexis & ultrasound phacoemulsification (*n* = 66); Group-B femtosecond-laser assisted capsulorhexis and lens fragmentation (*n* = 67), employing the LenSx laser (Alcon Surgical, Ft. Worth, TX). All cases were evaluated for refraction, visual acuity, keratometry, tomography, pachymetry, endothelial cell counts, intraocular pressure, and type of intraocular lens (IOL) implanted. The groups were matched for age, gender, pre-operative vision metrics, and cataract grade, and were followed up to 1 year.

**Results:**

In group-A post-operative uncorrected distance visual acuity (UDVA) was 20/20 or better in 61.5 % and 20/25 or better in 78.5 % of the eyes. The femtosecond laser group-B had improved outcomes (*p* = 0.075 and *p* = 0.042, respectively): post-operative UDVA was 20/20 or better in 62.7 % of the eyes and 20/25 or better in 85.1 %.

Linear regression scatterplots of achieved versus attempted spherical equivalent had excellent regression coefficients (*r*^*2*^ = 0.983 in group-A and 0.979 in group-B). There were 75.2 % cases in group-A and 80.6 % in group-B (*p* = 0.8732) within ±0.50 D of targeted refractive equivalent. Slight trend of under-correction was noted in group-A. Average residual manifest cylinder in the toric subgroup-A was -0.50 D (95 % Limit-of-Agreement (LoA) = -0.78 D), and in toric subgroup-B -0.45 D (LoA = -0.45 D).

**Conclusions:**

Mean spherical equivalent refraction and visual acuity are comparable with laser cataract surgery compared with manual capsulorhexis & ultrasound phacoemulsification. Improved astigmatism correction may be among the benefits of femtosecond laser–assisted cataract surgery. Transient corneal edema may be a first day transient disadvantage in femtosecond laser–assisted cataract surgery.

## Background

Cataract removal surgery is considered one of the most safe and efficient procedures in all of medicine today. However, some limitations are noted [1]. Poor wound integrity and subclinical wound leaks from clear-cornea incisions have been associated with an increased rate of endophthalmitis [2–5]. The quest for smaller incisions and reduced energy has been continuous and also reported by our team [6, 7] yet the current procedure’s outcome largely depends on surgeon skills and experience. Inconsistent capsulorhexis (continuous curvilinear capsulotomy), intraocuLar lens (IOL) tilt and decentration as well as posterior capsule opacification may lead to residual refractive error and to compromised visual rehabilitation [8–14].

Over the past decades, continuous evolution and refinement in cataract surgery has led to increased accuracy and satisfactory refractive results. This, in turn, has led to an increase in patient expectations. In a benchmark study [15], researchers concluded that refractive outcomes in cataract surgery for normal eyes should be within ±0.50 D for 55 %, and within ±1.00 D for 85 % of the cases. Effective IOL position (ELP) variability has been identified among parameters responsible for the inconsistency of refractive outcomes [16].

The femtosecond laser-assisted cataract surgery offers a femto-second laser-assisted option to three basic steps in cataract surgery: corneal incisions, capsulotomy, and initial lens fragmentation. This is achieved by ultra-short (few hundred femtoseconds) laser pulses operating at 1,056 nm wavelength, focused in a similar fashion employed in the flap creation during laser in situ keratomileusis (LASIK) The difference is that the focused beam may be adjusted to reach not only the cornea (for the corneal incisions), but also deeper, within the crystalline lens. The procedure is safe and effective as conventional manual incision combined with ultrasound phacoemulsification may help to improve refractive predictability by reducing ELP variability [17–26].

Among the potential adverse effects associated with the use of femtosecond laser in cataract surgery are incision decentration; incomplete or interrupted capsulotomy, fragmentation, or corneal incision procedure; capsular tear; corneal abrasion or defect; pain; infection; bleeding; damage to intraocular structures; anterior chamber fluid leakage and anterior chamber collapse; and elevated intraocular pressure (IOP). Some of these effects are unique to the femtosecond-laser assisted option; however most of these adverse effects are also relevant in the standard ultrasound phacoemulsification.

This comparative study aims to elucidate the clinical and refractive outcomes of this emerging clinical entity.

## Methods

This is a single-center, comparison, single-intervention case series, and prospective data evaluation of 133 consecutive cases subjected to cataract surgery (with or without corresponding astigmatism correction).

This study received approval by the Ethics Committee of our Institution (LaserVision Clinical & Research Eye Institute), and adhered to the tenets of the Declaration of Helsinki. Signed informed consent was obtained from each patient at the time of the first study visit.

Two groups were formed, based on the primary method of cataract surgery. In group-A (Phaco), manual corneal incisions, capsulorhexis and ultrasound phacoemulsification were employed (*n* = 66 eyes); the Constellation (Alcon Surgical, Ft. Worth, TX) microsurgical system was employed for ultrasound phacoemulsification and cataract removal. The term ‘small incision’ refers to the use of a sutureless, 2.75 mm clear-cornea main incision in the superior quadrant.

In group-B, the LenSx (Alcon) femtosecond laser (fully described in [1] and [18]) was employed for the capsulorhexis, more accurately defined as circular laser assisted capsulotomy when performed by the femtosecond laser, and in-situ lens fragmentation (*n* = 67 eyes). The SoftFit patient interface (applanation cone) was used during docking. The Constellation system was employed to perform or complete the phacoemulsification in both groups. The decision to perform either manual capsulorhexis & ultrasound phacoemulsification or femtosecond-laser assisted procedure was based on random choice (randomization table) prior to the operation.

In both groups the AcrySof IQ IOL (Alcon) was implanted in the intracapsular bag. These one-piece IOLs consist of hydrophobic acrylic material with refractive index *n* = 1.55, and have 6.0 mm diameter optical zone, and biconvex aspheric design. The AcrySof IOLs are also available in toric versions T2 through T9, corresponding with increasing magnitude of astigmatic correction. In both groups optical biometry employing optical low-coherence reflectometry (OB820, Alcon, based on the LenStar by Haag-Streit, Switzerland) [2] was employed to calculate IOL power using the SRK-T formula, including toric components. Toric IOLs was attempted in all cases: The keratometric astigmatism values and axis, as defined by the Lenstar measurement, were added for each case in the Alcon toric IOL online calculator: www.acrysoftoriccalculator.com/asp. We used as default in these measurements a standard 2.75 mm main incision to be placed at 135 degrees, and a 1 mm paracentesis at 0 degrees, respectively. Also by default, we targeted a postoperative astigmatic target of up to -0.2 diopters at 180 degrees (slight with the rule astigmatism).

In both groups the post-operative regimen consisted of a combination of antibiotic and corticosteroid drops (dexamethasone and tobramycin), administered for four weeks. Further surgical details are provided in previous publications [3–5]. All operations were performed by the same surgeon (AJK), and in all procedures the pre-defined placement of the main and side incision were marked and adhered to.

### Inclusion criteria

Successful primary cataract removal and IOL intracapsular bag insertion cases with or without toric IOL use. Preoperative myopia up to -14.00 D, hyperopia of up to +8.00 D, and up to -5.00 D of astigmatism. Pre-operative endothelial cell density (ECD) of more than 1,700 cells/mm^2^.

### Exclusion criteria

Clinically significant corneal abnormalities including basement membrane dystrophy and endothelial dystrophy, significant superficial punctuate keratitis, poorly dilating pupil in relation to the intended capsulotomy diameter, or other abnormalities that in the surgeon’s opinion (AJK) would negatively affect the safe outcome of the procedure, such as any sign of corneal disease and corneal scar within the optical zone. No cases were included in the study if pre-operative macular degeneration was noted prior to the procedure to avoid influence on visual acuity and refraction data. Additionally no cases that had prior cornea surgery (e.g. laser-vision correction) were included.

### Data collection and analysis

All cases were evaluated pre-operatively and up to one-year postoperatively (one-day, one-week, one-month, three-, six- and twelve-months). The following patient data were recorded: Age, gender, eye laterality, visual acuity, refraction (spherical equivalent; refractive astigmatism), keratometry (flat and steep simulated keratometry), ECD, central corneal thickness (CCT), and IOP. Uncorrected distance visual acuity (UDVA) and best-spectacle corrected distance visual acuity (CDVA) were evaluated using the Snellen chart. Refraction was assessed via manifest phoropter examination. ECD was measured by specular microscopy (FA-3709, Konan Medical, Irvine, CA). Corneal thickness maps were obtained by anterior-segment optical coherence tomography (OCT, RtVue-100, Optovue, Fremont, CA) [6]. Keratometry was assessed via Scheimpflug-imaging topometry (Pentacam, Oculus Optikgeräte GmbH, Wetzlar, Germany). IOP was assessed by Goldmann applanation tonometry. The associate Optometry staff collected data during the scheduled patient visits, not aware, at the time of the data collection, of the potential future use of these data for the purpose of the present analysis.

Surgery-specific data were also evaluated, including phacoemulsification time (expressed in seconds, s), and cumulative dissipated energy (CDE) (expressed in Joules, J). Specifically, CDE was provided by the mean ultrasound phaco power (W) × phacoemulsification time (s). This technique errs on the side of ultrasound phacoemulsification (phaco group-A) as the lens is partially fragmented in-situ prior to ultrasound application in the femtosecond laser group-B. IOL sphere and cylinder power implanted (expressed in D, diopters), and target spherical and cylinder refraction (D) were also among the data evaluated. These measurements were performed at 1, 3, 6 and 12 months postoperative in all cases to account for proper wound healing and refraction stabilization.

Additional pre-operative data evaluated included corneal topography based on Placido-ring topography (Vario, Alcon/WaveLight, Ft. Worth, TX, based on the K4 Topographer by Oculus, Germany), [27] corneal tomography utilizing Scheimpflug imaging (Oculyzer II, Alcon, based on the Pentacam HD by Oculus, Germany), [28] as well as retinal imaging employing Fourier-domain OCT (RtVue-100, Optovue). In addition, the Lens Opacity Classification System III (LOCS III) [7, 8] was employed for cataract grading.

Refractive data are presented in the standard refractive outcome format [9]. Descriptive statistics and analyses were performed by Minitab version 16.2.3 (MiniTab Ltd., Coventry, UK). Statistical significance was assessed using student t-tests. *P*-values less than 0.05 were indicative of statistically significant differences.

## Results

The 66 eyes included in group-A (Phaco) belonged to 37 female and 29 male patients; 32 eyes were right (OD) and 34 left (OS). Mean patient age at the time of the operation was 69.92 ± 11.73 (51 to 88) years. All cases were completely evaluated until the three-month interval; 60 were available for the six-month interval, and 59 were available for the final, one-year follow-up. The 67 eyes in group-B (Femto-laser) belonged to 40 female and 27 male patients; 34 eyes were right (OD) and 33 left (OS). Mean patient age at the time of the operation was 67.33 ± 11.99 (40 to 85) years. All cases were completely evaluated until the three-month interval; 62 were available for the six-month interval, and 58 were available for the final, one-year follow-up. Demographic and pre-operative characteristics by treatment group are reported in Table 1.

**Table 1:** Demographic, pre-operative baseline characteristics, and surgical data by treatment group

	Phaco group-A	Femto-second laser group-B	*p*-value
Eyes analyzed (n):	66	67	
Right: Left	32 (48.5 %): 34 (51.5 %)	34 (50.7 %): 33 (49.3 %)	-
Female: Male	37 (56.1 %): 29 (43.9 %)	40 (59.7 %): 27 (40.3 %)	-
Age (years)	69.92 ± 11.73 (51 to 88)	67.3 ± 11.99 (40 to 85)	0.168
UDVA (decimal)	0.28 ± 0.23 (0.01 to 0.80)	0.30 ± 0.24 (0.01 to 0.80)	0.821
CDVA (decimal)	0.68 ± 0.27 (0.01 to 1.00)	0.69 ± 0.22 (0.01 to 1.00)	0.78
Sphere (D)	−1.96 ± 4.72 (-15.75 to +9.125)	−1.62 ± 4.33 (-14.25 to +7.125)	0.65
Cylinder (D)	−1.07 ± 0.88 (-4.00 to 0.00)	−0.96 ± 0.8 (-5.00 to 0.00)	0.781
Flat keratometry (D)	42.99 ± 1.29 (39.29 to 45.18)	43.32 ± 1.26 (41.25 to 46.59)	0.821
Steep keratometry (D)	44.07 ± 1.25 (41.24 to 46.83)	44.16 ± 1.54 (41.82 to 48.54)	0.982
IOP (mmHg)	15.48 ± 3.78 (8 to 28)	14.95 ± 2.96 (9 to 22)	0.691
Target Sphere (D)	−0.17 ± 0.51 (-2.50 to 0.00)	−0.05 ± 0.25 (-1.50 to 0.00)	0.075
Target Cylinder (D)	0.18 ± 0.24 (0.00 to 0.50)	0.18 ± 0.24 (0.00 to 0.50)	0.923
Axial Length (mm)	24.72 ± 2.72 (20.20 to 36.30)	24.09 ± 1.75 (21.42 to 30.00)	0.834
LOCS classification	2.59 ± 0.94 (1 to 4)	2.37 ± 1.18 (1 to 4)	0.753
Phacoemulsification time (s)	160 ± 58 (99 to 225)	77 ± 39 (25 to 145)	0.015
Cumulative dissipated energy (J)	5.3 ± 2.8 (1.9 to 7.5)	2.4 ± 2.2 (0.9-5.4)	0.024
IOL Sphere power implanted (D)	18.95 ± 6.22 (2.00 to 36.00)	20.10 ± 5.01 (4.00 to 28.00)	0.643
# of toric IOL implanted	27/66	25/67	-
IOL Cylinder Power implanted (D)	1.87 ± 0.70 (0.00 to 3.80)	1.38 ± 0.72 (0.00 to 3.00)	0.235

*Abbreviations: UDVA=* uncorrected distance visual acuity, *CDVA=* corrected distance visual acuity, *IOP=* intraocular pressure, *LOCS=* Lens Opacity Classification System, *IOL=* intraocular lens. Results are presented in the form: average ± standard deviation (minimum to maximum)

### Pre-operative baseline data

Pre-operatively, average refractive error in group-A was sphere -1.96 ± 4.72 (-15.75 to +9.125) D and cylinder -1.07 ± 0.88 (-4.00 to 0.00) D, and in group-B sphere -1.62 ± 4.33 (-14.25 to +7.125) D and cylinder -0.98 ± 0.80 (-5.00 to 0.00) D. Pre-operative average UDVA in group-A was 0.28 ± 0.23 (0.01 to 0.80) and CDVA 0.68 ± 0.27 (0.01 to 1.00), reported decimally. In group-B, average pre-operative UDVA was 0.30 ± 0.24 (0.01 to 0.80) and CDVA 0.69 ± 0.22 (0.01 to 1.00).

Pre-operative keratometry in group-A was along the flat meridian 42.99 ± 1.29 (39.29 to 45.18) D and along the steep meridian 44.07 ± 1.25 (41.24 to 46.83) D, while in group-B flat keratometry was 43.32 ± 1.26 (41.25 to 46.59) D and steep keratometry 44.16 ± 1.54 (41.82 to 48.54) D.

Lens Opacity Classification System grading was 2.59 ± 0.94 (1 to 4) for group-A and 2.37 ± 1.18 (1 to 4) for group-B. Pre-operative IOP in group-A was 15.48 ± 3.78 (8 to 28) mmHg and in group-B 14.95 ± 2.96 (9 to 22) mmHg. In group-A target spherical refraction was -0.17 ± 0.51 (-2.50 to 0.00) D and target cylinder 0.18 ± 0.24 (0.00 to 0.50) D; axial length was 24.72 ± 2.72 (20.20 to 36.30) mm. IOL spherical power implanted in group-A was 18.95 ± 6.22 (2.00 to 36.00) D, and cylinder power 1.87 ± 0.70 (0.00 to 3.80) D. In group-B, target spherical refraction was -0.05 ± 0.25 (-1.50 to 0.00) D and target cylinder 0.18 ± 0.24 (0.00 to 0.50) D; axial length was 24.09 ± 1.75 (21.42 to 30.00) mm. IOL spherical power implanted in group-B was 20.10 ± 5.01 (4.00 to 28.00) D, and cylinder power 1.38 ± 0.72 (0.00 to 3.00) D.

The two groups were matched in all pre-operative and surgical planning aspects as none of the above-mentioned data sets indicated any statistically significant difference between the two groups.

### Surgical data

Phacoemulsification time in group-A was 160 ± 58 (99 to 225) s, while in group-B 77 ± 39 (25 to 145) s. CDE employed for phacoemulsification in group-A was 5.3 ± 2.8 (1.9 to 7.5) J and in group-B 2.4 ± 2.2 (0.9-5.4) J. Both of these parameters had a statistically significant difference (*p* <0.001).

**Fig. 1:**
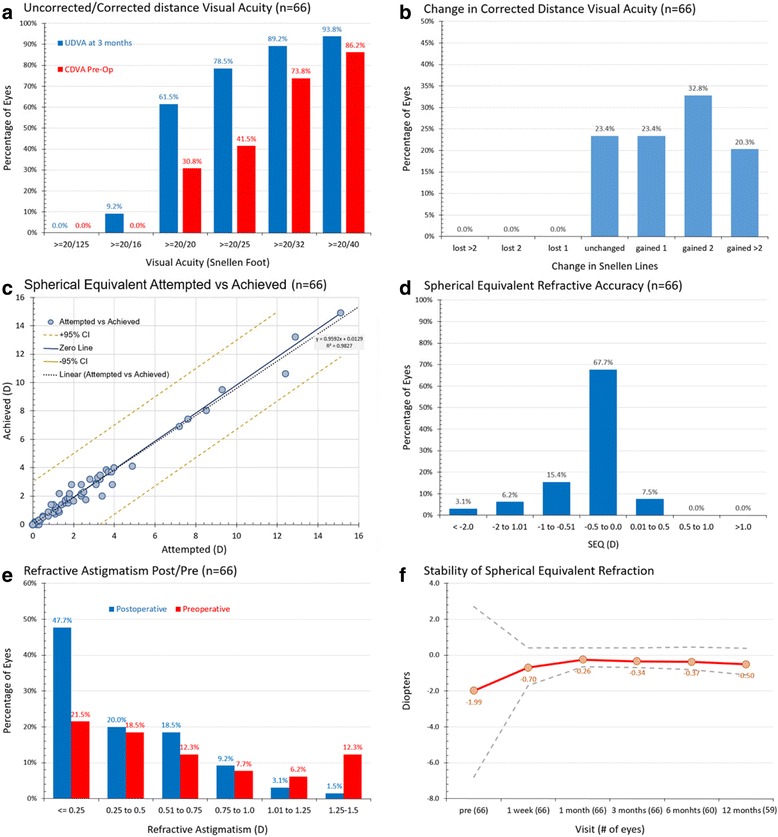
Standard graphs for reporting refractive surgery outcomes for group-A (Phaco). UDVA, uncorrected distance visual acuity; CDVA, corrected distance visual acuity

**Fig. 2:**
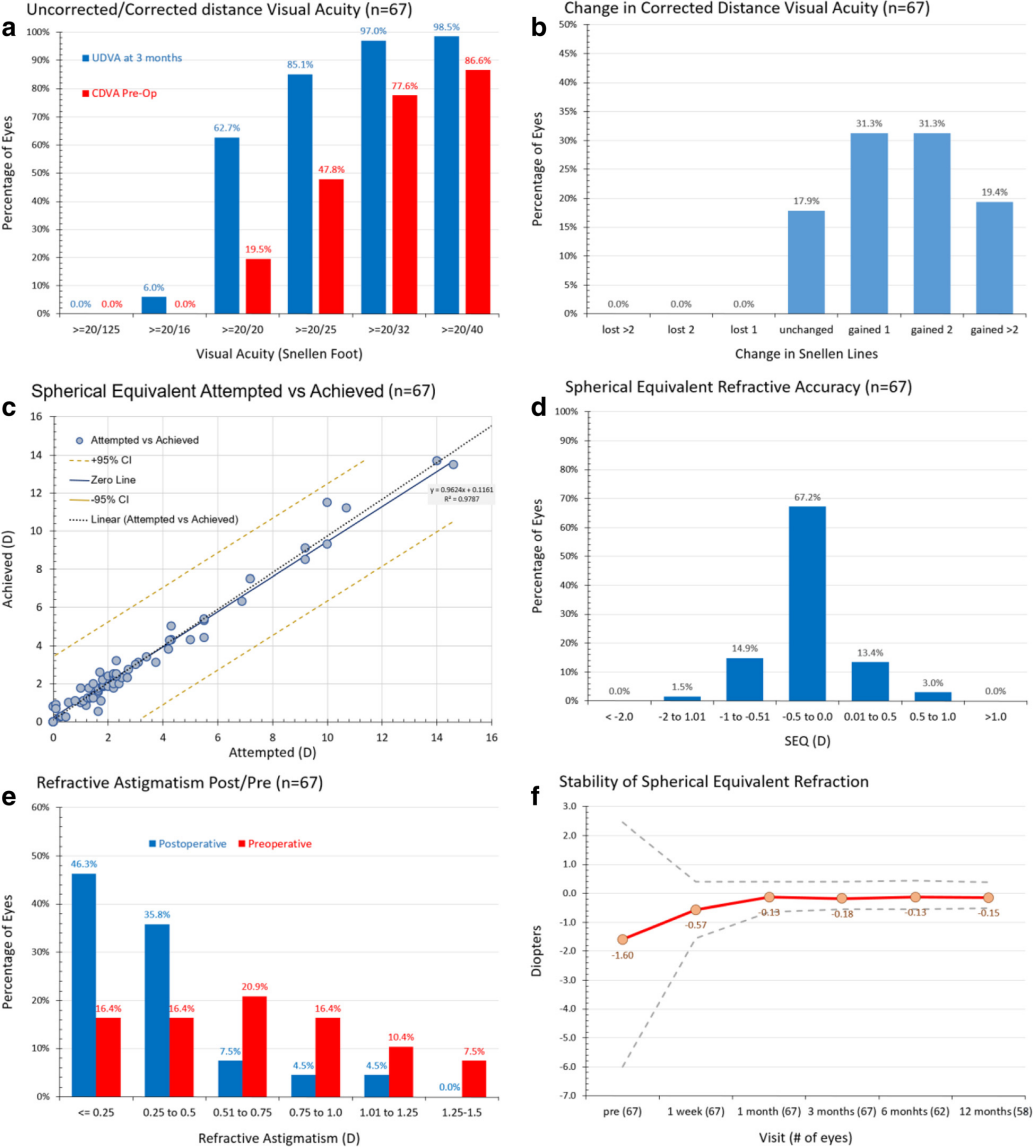
Standard graphs for reporting refractive surgery outcomes for group-B (LenSx). UDVA, uncorrected distance visual acuity; CDVA, corrected distance visual acuity

Standardized-form one-year postoperative refractive outcomes for phaco group-A and femtosecond group-B are presented in Figs. 1 and 2, respectively.

### Uncorrected visual acuity outcome and stability

As shown in the monocular distance visual acuity outcome results in the phaco group-A (Fig. 1a, top left), 61.5 % of the eyes had post-operative UDVA 20/20 Snellen (1.0 decimal) or better, and 78.5 % had 20/25 Snellen (0.8 decimal) or better. In the femtosecond laser group-B (Fig. 2a, top left), 62.7 % of the eyes had post-operative UDVA better than 20/20 (1.0 decimal), and 85.1 % had better than 20/25 (0.8 decimal).

### Efficacy of corrected visual acuity

The gain-loss data (change in Snellen lines of CDVA) indicate that in the phaco group-A (Fig. 1b, top right), 23.4 % of the eyes were unchanged, 23.4 % gained one, 32.8 % gained two, and 20.3 % gained more than two Snellen lines. In the femtosecond laser group-B (Fig. 2b, top right), 17.9 % of the eyes were unchanged, 31.3 % gained one, 31.3 % gained two, and 19.4 % gained two or more lines. No eye lost any line in either group.

### Refractive predictability and accuracy

Predictability results are illustrated in Figs. 1c (group-A) and 2c (group-B) (both middle row, left), in which the achieved spherical equivalent versus attempted spherical equivalent (in D) is plotted in the form of a linear regression scatterplot. Both plots have excellent regression coefficients (*r*^*2*^ = 0.983 in group-A and 0.979 in group-B). There were 3 overcorrected (by 0.5 D) and 6 under-corrected cases in group-A, and 7 over-corrected and 9 under-corrected cases in group-B; there were 3 under-corrected (by 1.0 D) cases in group-A and only 1 over-corrected (by 1.0 D) case in group-B.

Refractive accuracy results are illustrated in Figs. 1d (group-A) and 2d (group-B) (both middle row, right). Within ±0.5 D there were 75.2 % of the eyes in group-A and 80.6 % in group-B (*p* =0.8732). There was a slight trend of under-correction noted in group-A: 24.7 % had myopic spherical equivalent vs. 7.5 % had slightly hyperopic, between 0.00 and +0.50 D. The results were more balanced in the femtosecond laser group-B: 16.4 % had slightly myopic spherical equivalent and 16.4 % slightly hyperopic.

Figures 1e and 2e (bottom row, left) display the postoperative refractive astigmatism within intervals of 0.50 D, representing the accuracy of cylinder correction. In the phaco group-A, 67.7 % of the cases had post-operative cylinder less than 0.50 D, while in the femtosecond group-B, 82.1 %.

### Refractive stability

Refractive stability is demonstrated by the manifest refractive spherical equivalent (MRSE) as followed during the 1-, 3-, 6-, and 12-month post-operative visits (Figs. 1f and 2f, bottom row, right). The 12-month postoperative mean MRSE was -0.51 ± 0.37 D in group-A and -0.16 ± 0.13 D in group-B. These findings indicate increased refractive stability and accuracy of the femtosecond laser group-B in comparison to the group (*p* =0.037).

### Longitudinal corneal thickness changes

**Fig. 3:**
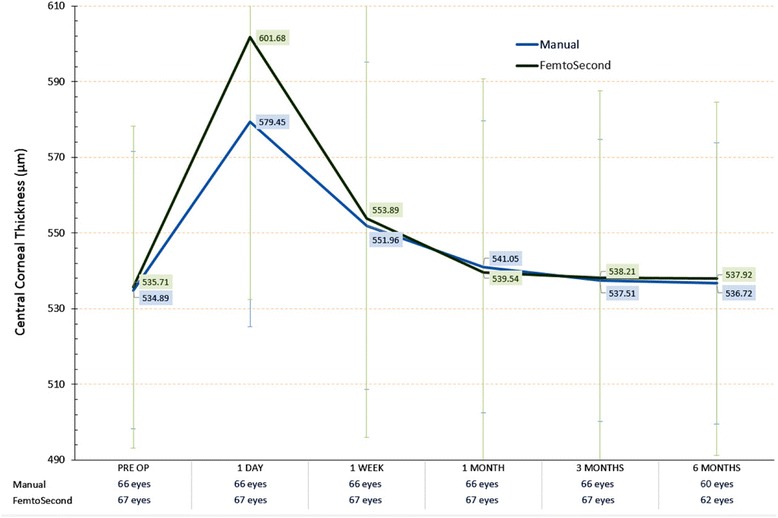
Central Corneal Thickness as monitored pre-operatively and post-operatively (1 day, 1 week, 1 month, 3 months, and 6 months)

The longitudinal changes in central corneal thickness were evaluated with OCT pachymetry measurements pre-operatively, 1 day, 1 week, 1 month, 3 months, and 6 months postoperatively. Results are illustrated in Fig. 3 and analytically presented in Table 2. We note a near-term transient corneal swelling, with a statistically significant difference (*p* <0.01) between the two groups: the one-day (+65.97 μm) and one-week (+18.18 μm) average corneal thickness increase in the femtosecond laser group-B was larger compared to group-A’s one-day (+44.56 μm) and one-week (+17.07 μm). Deturgescence was noted after one-month in both groups, indicating the transient nature of this post-operative corneal swelling. After the one-month interval, the differences in average central corneal thickness between as well as in comparison to baseline in both groups were not statistically significant.

**Table 2:** Pre-operative and post-operative (1 day, 1 week, 1 month, 3 months, and 6 months) central corneal thickness measurements in the two groups of study

	Pre-Op	1 day	1 week	1 month	3 months	6 months
Group-A	Phaco					
Average	534.89	579.45	551.96	541.05	537.51	536.72
St Dev	36.68	54.23	43.2	38.54	37.22	37.14
Min	445	480	469	463	450	448
Max	647	720	714	692	654	650
Difference to baseline	-	44.56	17.07	6.16	2.62	1.83
Group-B	Femto second					
Average	535.71	601.68	553.89	539.54	538.21	537.92
St Dev	42.49	69.22	57.98	51.15	49.43	46.6
Min	465	520	474	470	465	462
Max	654	766	712	654	654	654
Difference to baseline	-	65.97	18.18	3.82	2.5	2.21
*p*-value (paired test group-A to group-B)	0.883	0.045	0.815	0.735	0.855	0.882

Δ indicates difference to pre-operative baseline. All units are reported in μm. *P*-values correspond to comparison between groups for the same visit measurements

### Endothelial cell density

**Fig. 4:**
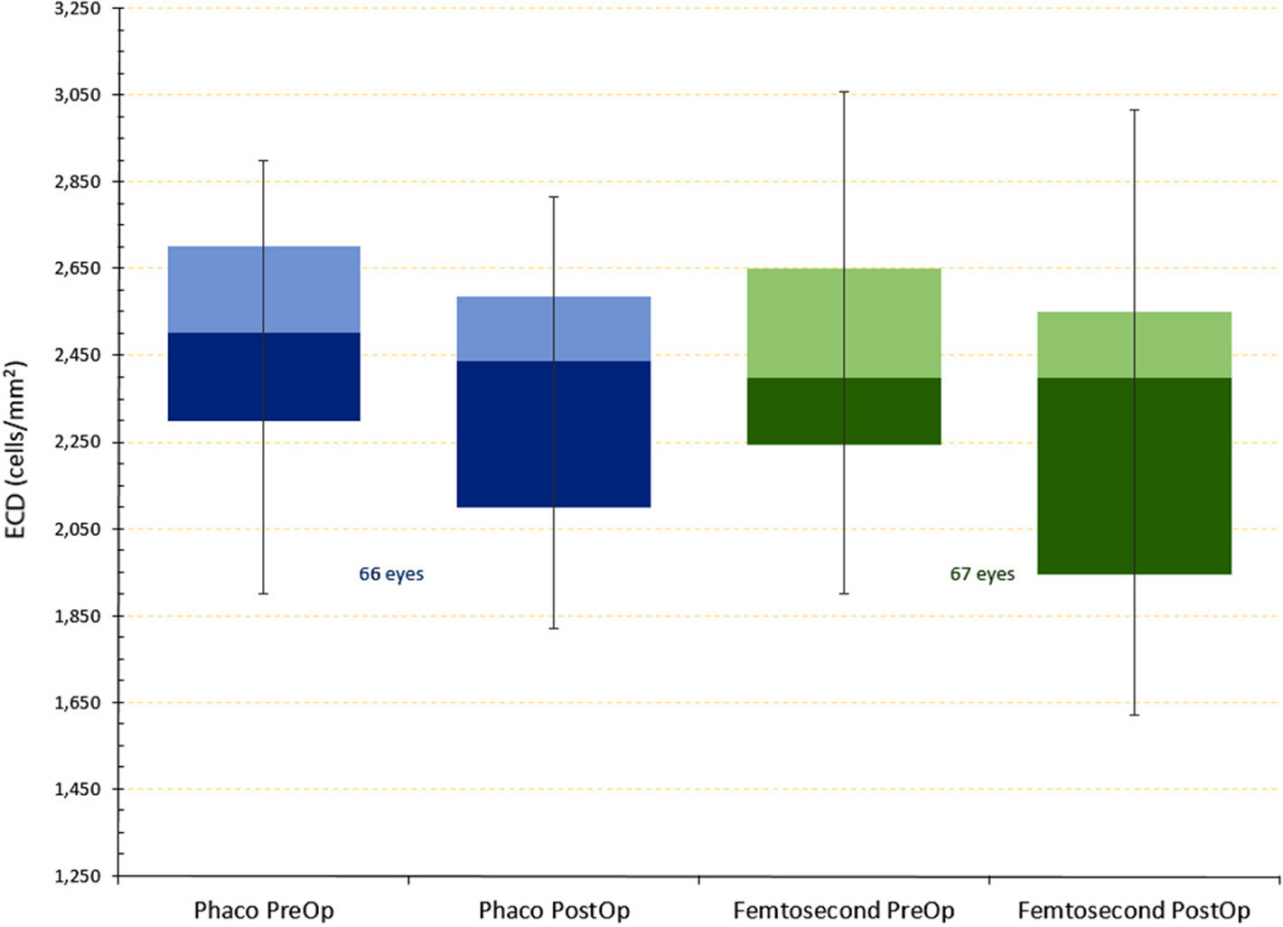
Endothelial cell counts as monitored pre-operatively and 3 months post-operatively

ECD measurements were conducted with specular microscopy pre-operatively and three months post-operatively. Results are illustrated in Fig. 4 and analytically presented in Table 3. The femtosecond-assisted group-B indicated a slightly increased drop of ECD in comparison to group-A (-6 % versus -3 %). None of the changes noted were statistically significant, however.

**Table 3:** Endothelial cell density (reported in cells/mm^2^). Results for the two groups, Phaco group-A and femtosecond-laser assisted group-B

		*Pre-Op*	*Post-Op*	*Δ(ECD reduction)*	*p-value*
*Phaco*	Average	2,428.59	2,348.12	−80.47 (-3 %)	0.1293
	St Dev	357.58	286.63		
	Min	1,700	1,821		
	Max	2,900	2,717		
*Femtosecond*	Average	2,481.27	2,325.33	−155.93 (-6 %)	0.0873
	St Dev	376.40	487.79		
	Min	1,900	1,520		
	Max	3,456	3,100		

### Toric IOL evaluation

**Fig. 5:**
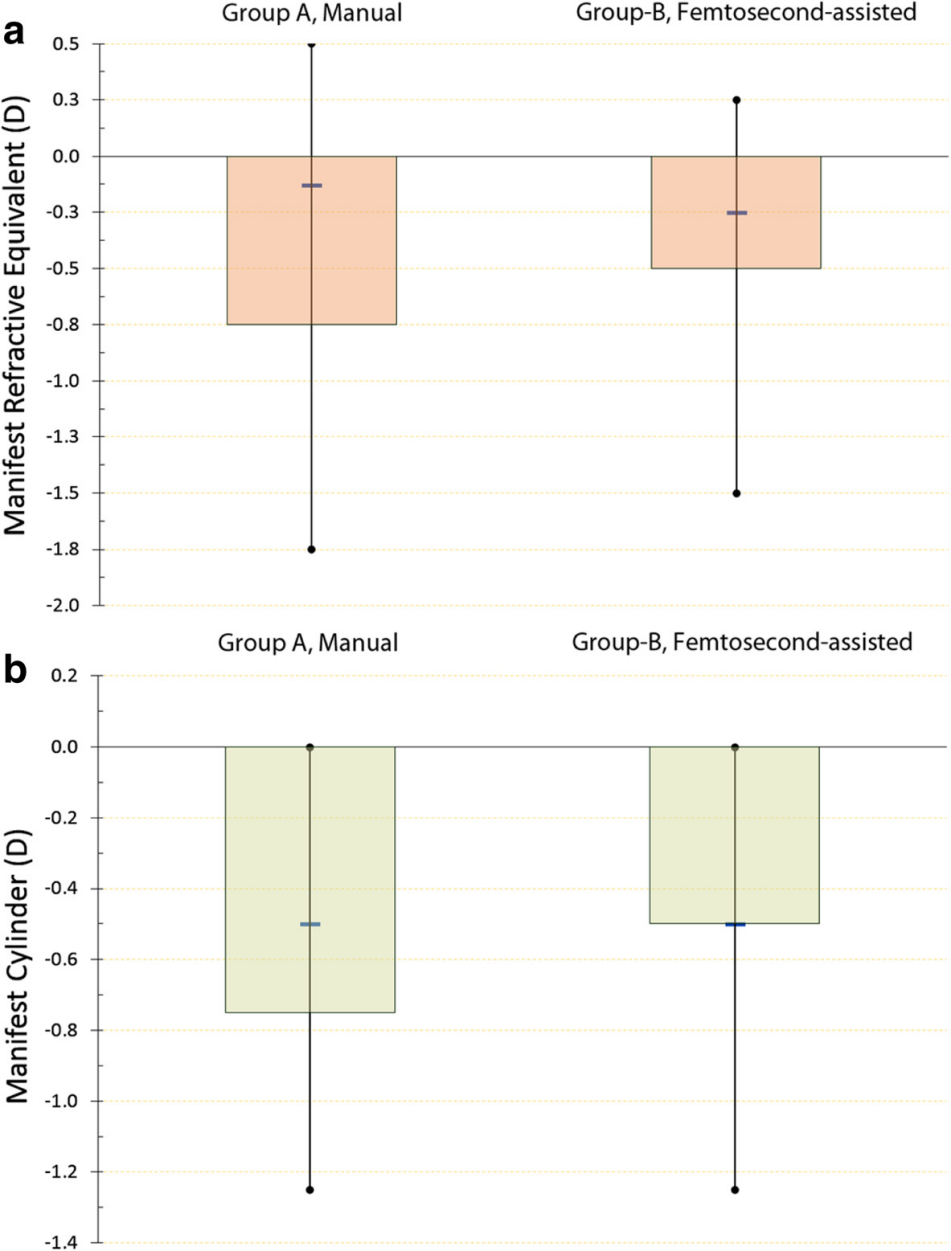
Comparative refractive outcomes for the toric phaco subgroup-A (*n* = 27) and femtosecond-assisted subgroup-B (*n* = 25). From top to bottom, **a**: Manifest refractive spherical equivalent (D), and **b**: Manifest Cylinder (D)

We further evaluated the refractive outcomes of the subgroups within each group in which toric IOLs were implanted. The AcrySof T2 up to T4 was employed in 26/66 cases in group-A, and in 24/67 cases in group-B. There was one T6 (3.75D) in group-A, and one T5 (3.0 D) in group-B. Average cylinder power implanted in group-A was 1.94 ± 0.60 (1.00 to 3.75) D and 1.60 ± 0.47 (1.00 to 3.00) D in group-B. Comparative results are illustrated in Fig. 5.

Preoperative MRSE in toric subgroup-A was -2.17 ± 4.95 D, and in the toric subgroup-B -1.90 ± 4.38 D (*p* =0.18). Three-month postoperative residual manifest cylinder for toric phaco subgroup-A was -0.39 ± 0.95 D and for the toric subgroup-B -0.27 ± 0.19 D (*p* =0.131).

Preoperative manifest cylinder in toric subgroup-A was -1.05 ± 0.89, and in toric subgroup-B, -0.96 ± 0.80 (*p* =0.23). Three-month postoperative residual manifest cylinder for toric subgroup-A was -0.53 ± 0.38 D, and in toric femtosecond laser subgroup-B -0.41 ± 0.24 D (*p* =0.075).

## Discussion

Cataract surgery, although initially employed to simply remove the opaque crystalline lens, has recently been considered a ‘refractive’ procedure, as the refractive outcome can be safely planned and refractive outcomes are increasingly predictable. This expected optimal refractive outcome might significantly improve the quality of everyday life. Patients and clinicians’ expectations dictate the least amount of post-operative astigmatism and sphere. Pre-operative refraction accuracy in cataract patients may be elusive depending on the degree and/or type of opacification [29, 30]. It nevertheless constitutes a real clinical metric that may significantly affect visual function nowadays and may serve as one of the main symptoms, and/or main indications for cataract extraction. Thus we included in this study pre- to post-operative refraction comparisons in order to clearly document the potential for refractive efficacy of modern cataract surgery with either of the two techniques studied.

The advent of femtosecond laser technoligies are reported to project potential benefits in cataract surgery [27, 28, 31–35]. The LenSx employs a solid-state amplified laser operating in the near infrared; the beam is directed by means of an integrated video microscope and live optical coherence tomography (OCT) imaging. Similar to femtosecond lasers in LASIK, the LenSx requires stable corneal reference interface for precise depth control during the procedure. For this purpose, docking and focusing is facilitated via a disposable applanating patient interface, the SoftFit applanation cone [36].

The projected improvement claimed in corneal incision and capsular centration and dimensions precision, as well as the reduction of total ultrasound energy required for lens nucleus breakdown in cases with in-situ laser lens fragmentation prior to the phacoemulsification stage may potentially improve refractive outcomes and enhance safety for patients in comparison to traditional phacoemulsification [37]. Another potential benefit is derived from exact positioning and dimensioning of the anterior capsular opening, which may also help reduce IOL decentration and tilt [38].

In this study, we comparatively evaluated two large groups of eyes subjected to the established manual capsulorhexis & ultrasound phacoemulsification in comparison with the femtosecond laser-assisted cataract surgery. The refractive results indicate that in all aspects, in many of them by a statistically significant margin, the femtosecond laser group-B offered improved results in comparison to the manual group-A. Further, the toric IOL subgroup study indicated improved manifest sphere and cylinder for the femtosecond-assisted procedure.

Cataract surgery is known to induce, to some degree, corneal endothelial cell loss. It has been suggested that the femtosecond-laser may reduce the amount of required ultrasound energy, a factor known to be directly related to endothelial cell loss. The results in our study indicate that in the femtosecond laser group-B the mean ultrasound energy required for phacoemulsification was reduced by 65 % and the phacoemulsification time by 52 % (both *p* < .001). The reduced phacoemulsification time and dissipated energy (CDE) may correlate to reduced effect to the endothelial cells [39]. The reports in the peer-review literature are, however, inconclusive: some studies report adverse effect to the corneal endothelial cells by femto-second laser incisions [40], while other studies demonstrate a rather comparable endothelial cell loss between manual ultrasound phacoemulsification and femto-second assisted cataract surgery [41]. Our study did not indicate any statistically significant difference in endothelial cell density loss either, despite the reduced time and CDE levels.

The corneal injury after phacoemulsification may be attributed to several factors: surgical instrument-induced mechanical damages, lens fragments contacting the endothelium, trauma by energy dissipated close to the endothelium, and irrigating solution volume used and/or turbulence in the anterior chamber. We must, however, acknowledge that the reported CDE is derived from the ‘ultrasound’ part only. In the femtosecond-assisted cases, infrared laser energy is also transcending through the endothelium, during the capsulorhexis and initial lens fragmentation stages. The difference, though is that the non-ionizing infrared wavelength (1,050 nm) is far above the threshold for inducing cell damage (which lies in the UV region); in addition, the infrared femtosecond laser beam transcending the cornea towards the crystalline lens has not yet reached focus, thus lacking the potential to induce cell damage at the layer of the endothelial cells [42].

The aspect of corneal swelling following femtosecond-laser assisted cataract surgery is also not conclusive in the peer-review literature. While transient cornea edema following manual-incision cataract surgery is common [43] studies with a femtosecond laser for cataract surgery and Scheimpflug-imaging pachymetry [44] indicated reduced corneal swelling in the femtosecond laser group; our results, although on a different laser and different pachymetry imaging device, indicate increased transient corneal edema in the femtosecond laser group. We have reported that the increased axial resolution of the OCT pachymetry in comparison to Scheimpflug imaging [45] may offer a more detailed and valid pachymetric transient analysis in this case.

The data in this study indicate that femtosecond laser-assisted cataract surgery is at least as effective as manual ultrasound phacoemulsification at achieving emmetropia. In addition, it appears to provide improved refractive outcomes in the subgroup of toric intraocular implantation cases possibly through improved effective lens position.

## Conclusions

Femtosecond laser–assisted cataract surgery is as safe and effective as manual incision & ultrasound phacoemulsification cataract surgery. Mean spherical equivalent refraction and visual acuity are comparable. Improved astigmatism correction may be among the benefits of femtosecond laser–assisted cataract surgery. Transient corneal edema delaying visual rehabilitation by a day or so was noted in femtosecond laser–assisted cataract surgery.
